# Case report: A novel truncating variant of *BCL11B* associated with rare feature of craniosynostosis and global developmental delay

**DOI:** 10.3389/fped.2022.982361

**Published:** 2022-10-06

**Authors:** Xuemei Zhao, Bingbing Wu, Huiyao Chen, Ping Zhang, Yanyan Qian, Xiaomin Peng, Xinran Dong, Yaqiong Wang, Gang Li, Chenbin Dong, Huijun Wang

**Affiliations:** ^1^Center for Molecular Medicine, Children’s Hospital of Fudan University, Shanghai, China; ^2^Department of Plastic Surgery, Children’s Hospital of Fudan University, Shanghai, China

**Keywords:** *BCL11B*, truncating variation, pediatrics, craniosynostosis, developmental delay

## Abstract

Craniosynostosis is a premature fusion of cranial sutures, resulting in abnormally shaped skull and brain development disorder. The description of craniosynostosis in patients with *BCL11B* mutations is rare. Here, we firstly report a 25-month-old Chinese boy with a novel frameshift variant in *BCL11B* gene. The patient was identified c.2346_2361del by whole-exome sequencing and was confirmed to be *de novo* by parental Sanger sequencing. This patient presented clinical phenotype of craniosynostosis as well as global developmental delay. He had a small mouth, thin upper lip, arched eyebrows, a long philtrum, midfacial hypoplasia and craniosynostosis. Brain MRI showed brain extracerebral interval and myelination changes, and brain CT with 3D reconstruction showed multi-craniosynostosis. Our study expands the clinical phenotypes of patients with *BCL11B* gene mutation, and our findings may help guide clinical treatment and family genetic counseling.

## Introduction

Craniosynostosis is the premature fusion of one or more of the calvarial sutures. The incidence of craniosynostosis is estimated to be 1 in 2000-2500 live births (1). In the cases of syndromic craniosynostosis, a series of clinical phenotypes, such as skull expansion, midfacial hypoplasia, increased intracranial pressure and dysmorphologies, are observed (2). The major etiology of craniosynostosis includes varieties of factors including genetic and environmental factors (3, 4). Moreover, genetic factor has been recognized as a non-negligible etiology of craniosynostosis. In a 13-year birth cohort study, one-quarter of patients with craniosynostosis could be identified as having a genetic cause (4). An early genetic diagnosis can provide valuable information for intervention. For the treatment of craniosynostosis, patients need long-term strategies, including surgery such as open cranial reconstruction, minimally invasive strip craniectomy with spring implantation, cranial distraction and a postoperative molding helmet (1).

The transcription factor *BCL11B* (B Cell Leukemia 11B), encoding Cys2-His2 zinc-finger protein transcription factor, is located on chromosome 14q32.2. BCL11B is broadly expressed and has critical functions in regulating multiple systems including the central nervous system, immune system, and skin (5, 6). BCL11B contains three types of functional structures: six C2HH zinc-finger binding domains (ZnF1-6), one C2HC zinc-finger binding domain, and a NuRD interacting domain (7). The first report of *BCL11B* as a potential disease gene was in an infant bearing a *de novo BCL11B* missense mutation with severe combined immunodeficiency, craniofacial and dermal abnormalities, and the absence of corpus callosum (8). Currently, 20 patients have been reported to harbor *BCL11B* pathogenic variants, The major clinical features include severe combined immunodeficiency, neurological disorders, dermal defects, congenital diaphragmatic hernia, and craniosynostosis. Recently, Goos et al. reported a *de novo* missense mutation in a patient with unilateral coronal suture craniosynostosis (9). However, it is rare about the description of craniosynostosis in patients with *BCL11B* mutations.

Here, we report a patient with a novel *de novo* frameshift variant exhibiting the combinational phenotype of craniosynostosis and developmental delay. Meanwhile, we review the clinical and genetic features of craniosynostosis patients with *BCL11B* mutations, which further delineate the phenotype and genotype of such patients.

## Materials and methods

### Whole exome sequencing and Sanger sequencing

Genomic DNA was extracted from peripheral blood using a whole blood genomic DNA extraction kit (Qiagen, German) according to the manufacturer’s protocol. DNA fragments were enriched for exome sequences using the IDT xGen Exome Research Panel v2 (Integrated DNA Technologies, Coralville, IA, USA). According to our previous reports, sequencing was performed on NovaSeq 6000 (Illumina, San Diego, USA) according to protocols (10). The data analysis followed the pipeline established by our team (11). PhenoPro, including phenotype-driven analysis and variant-driven analysis, was performed to prioritize the disease-causing genes of Mendelian diseases as described before (12). The pathogenicity was evaluated by the variation frequency, phenotypic coincidence with patients and the type of variation according to ACMG criteria (13). The variant of the *BCL11B* gene was confirmed by Sanger sequencing on an ABI 3,730 Genetic analyzer (Applied Biosystems, Foster City, CA, USA).

### *In silico* analysis

Nonsense-mediated mRNA decay (NMD) efficacy of the *BCL11B* frameshift was evaluated using online NMDective resource (14). The wildtype 3-D protein structure of BCL11B was analyzed by the neural network AlphaFold, a new online source to predict the protein domain (15). The mutated amino acid sequence was acquired on the Mutalyzer website. Then we used SWISS-MODEL to predict the truncated protein structure according to the protein template from AlphaFold. All the protein structures were visualized by PyMOL 2.3.0.

## Results

### Case presentation

**FIGURE 1:**
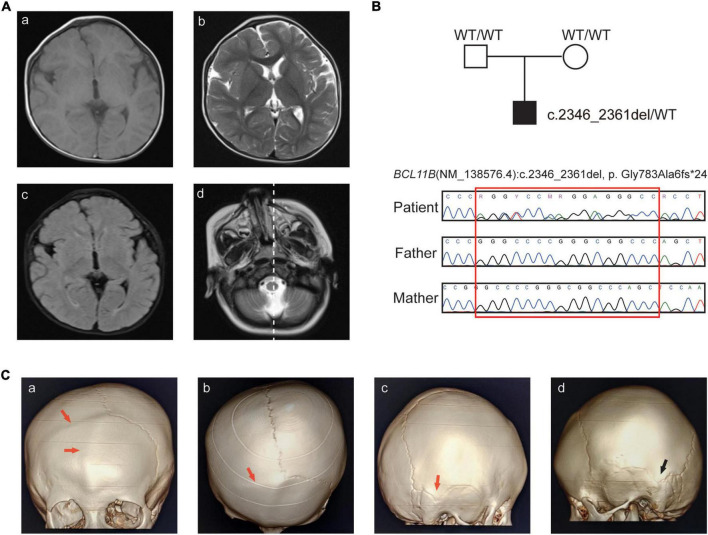
Clinical features and sequence analysis. **(A)** Brain MR images. Abnormal signals of the cornu occipitale. **(a)** T1WI hypodensities and the smaller right brain volume; **(b)** T2WI hyperintensity and the widening of the extracerebral space; **(c)** increased FLAIR signals; **(d)** right-skewed midfacial hypoplasia. **(B)** Pedigree of the family and Sanger sequencing analysis. **(C)** CT images with 3D reconstruction. **(a–b)** Frontal seam and right coronal seam, **(c)** right apex seam, **(d)** left apex seam. The abnormal seam fusions are indicated by the red arrow, and the normal seam is indicated by the black arrow.

The boy is the first child of healthy non-consanguineous parents. He visited our hospital at 12 months of age with global developmental delay and recurrent allergy. He was born after uneventful full-term pregnancy with birth weight of 3,650 g (0.60 SD), and birth length of 52.0 cm (1.49 SD). He was observed with plagiocephaly, his birth head circumference is 33.2 cm (−0.99 SD) (Supplementary Figure 1). He had midfacial hypoplasia, hypertelorism, arched eyebrows, a long philtrum and a small mouth (Table 1). His motor development milestones were delayed: he could not sit steadily at 14 months, he could walk with help at 18 months, and he could not clap his hands until 24 months, he could only say “baba” and “mama”. Brain MRI at the age of 13 months showed widening of the extracerebral interval, dysmyelination of the bilateral periventricular white matter, abnormal signals of the cornu occipitale and a reduced right-brain cerebral volume (Figure 1A and Table 1).

**TABLE 1:** Clinical features of six craniosynostosis patients with mutations in *BCL11B.*

Case	our patient	Goos et al.	Gaillard et al.	Gaillard et al.	Gaillard et al.	Gaillard et al.
cDNA	c.2346_2361del	c.7C > A	c.2000G > A	c.1744G > A	c.2018C > G	c.1265C > T
Protein	p.G783A fs*24	p.Arg3Ser	p.Gly667Glu	p.Gly582Ser	p.Pro673Arg	p.Pro422Leu
Type of variant	Frameshift	Missense	Missense	Missense	Missense	Missense
Origin of mutation	*De novo*	*De novo*	Maternal	Paternal	Maternal	Maternal
**Backgroud**						
Gender	Male	Male	Female	Male	Female	Male
Ethnicity	Chinese	Caucasus	Netherlands	Netherlands	Netherlands	Netherlands
Family history	None	None	None	None	None	None
**General**						
Intellectual disability	+	−	−	−	−	−
Speech impairment	+	−	+	Speech therapy required	−	−
Delay in motor development	+	−	+	Physical therapy required	−	−
Brain MRI	+	−	−	+	−	−
**Dysmorphic feature**						
Facial dysmorphism	A frontal plagiocephalic head shape, midfacial hypoplasia, a small mouth, thin upper lip, arched eyebrows and a long philtrum	A frontal plagiocephalic head shape, mild vertical orbital dystopia, a high philtrum, a short nose and narrow eyebrows	Slight ocular proptosis, hypertelorism, down slanting palpebral fissures, ptosis, arched eyebrows	Frontal bossing, down-slanting palpebral fissures, hypotelorism, mild webbing of the neck, and hyperpigmentation on the left shoulder	Mild vertical orbital dystopia	Thick alae nasi and mild retrognathia with an overbite
Anomalies of the hands	−	−	Brachydactyly (bilateral)	Short fifth digits (bilateral)	−	−
Anomalies of the feet	−	−	Syndactyly of the second and third toes (bilateral)	Syndactyly of the second and third toes (bilateral)	−	−
**Neurological**						
Hypotonia	+	−	+	−	−	−
Unstable gait	+	−	+	−	−	−
**Ophthalmologic**						
Refractive error	−	Hyperopia	Hypermetropia, astigmatism	Myopia, astigmatism	+ (Type unknown)	Not tested
Strabismus	−	−	−	−	−	Not tested
Other	−	−	Nystagmus	−	−	−
**Immune system function**						
IgE	↑	−	NR	−	−	−
Eosinophil	↑	−	NR	−	−	−
Frequent infection	−	−	+	−	−	−
Allergy/asthma	Allergy for dust mites, eggs, milk, wheat, and shrimp/crab	−	Asthma(light), allergy for dust mites and grass pollen	−	−	−
**Dental**						
Dental anomalies	−	−	−	−	−	Overbite
**Skull**						
Craniosynostosis	Synostosis of the frontal seam and right coronal suture	Synostosis of the right coronal suture	Sagittal suture craniosynostosis	Craniosynostosis of the sagittal suture and both lambdoid sutures,	Left-sided unicoronal craniosynostosis	Craniosynostosis of the sagittal suture

+, present; −, absent; ↑, the value is increased; NR, not reported.

Further examinations, including brain CT and blood immunophenotyping analysis, were undertaken at the age of 25 months. Skull radiographs and three-dimensional computed tomography (3D-CT) showed craniosynostosis of the frontal and right-coronal suture fusion, and skull asymmetry was observed, with the eye axis deviating to the right side (Figures 1A,C).

His immunophenotyping analysis results are shown in Supplementary Table 1 and Supplementary Figure 2. Notably, the number and percentage of eosinophil cells were increased to 910 cells/μl (normal range: 60-300 cells/μl) and 13.7% (normal range: 0.5-5%), respectively. The value of IgE in this patient was markedly increased to 944.91 IU/ml (normal range < 100 IU/ml).

### Genetic analysis

**FIGURE 2:**
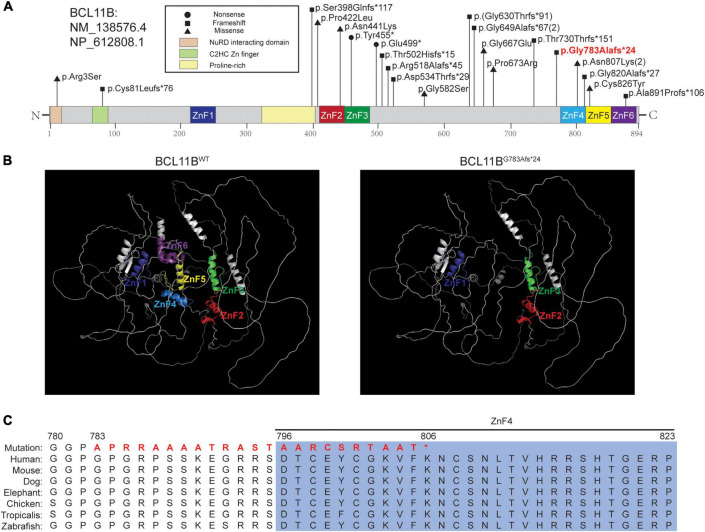
The pathogenic variant of *BCL11B* and the *in silico* prediction results. **(A)** The localization of variants reported in the literature and the variant in our patient. The variant in bold red font is that of the patient in this report. **(B)** Structures of BCL11B^WT^ analyzed by the AlphaFold network and BCL11B^G783Afs*24^ predicted by SWISS-MODEL. The ZnF1-ZnF6 domains are marked in the corresponding colors of the schematic representations. **(C)** Sequence alignment across species with amino acid residues was performed, and our patient’s mutated sequence is shown in bold red font.

WES was performed for the patient, and a novel heterozygous variant (NM_138576.4: c.2346_2361del, p.Gly783Ala6fs*24) of the *BCL11B* gene was detected. The variant was located on the exon4, just before the ZnF4 domain (Figure 2A) and was predicted to generate a truncated protein with a lack of ZnF4-6 (Figure 2B). The *BCL11B* gene variant was highly conserved and partly located in the ZnF4 domain (Figure 2C).

This frameshift variant was curated according to the ACMG criteria base on the following evidence: (i) NMDetective-A (score: 0.12) and NMDetective-B (score: 0) predicted the variant escaping NMD; the pLI (probability of being loss-of-function intolerance) score of *BCL11B* was 0.99, the observed/expected (o/e) ratio was 0.09, which suggests that *BCL11B* is highly possible of loss-of-function, PVS1_Strong; (ii) Sanger sequencing of parental samples confirmed that the variant was *de novo* (Figure 1B), and the phenotype was consistent with the *BCL11B* gene, PM6; (iii) the variant had not been recorded in gnomAD, the 1,000 Genomes database or our in-house database (43,000 individuals), PM2. (iv) we had submitted this variant to ClinVar database (VCV001184138.4) as pathogenic, and the lab of Institute of Human Genetics, University Hospital Muenster also submitted to ClinVar, with the evidence of PVS1 + PS2 + PS4, this same variant identified from a different patient. Therefore, the class of this variant was curated as pathogenetic.

We also reviewed currently reported 20 patients in literature. 18 patients carried 16 variations of the *BCL11B* gene, including ten frameshifts, four missense mutations and two nonsense mutations. The other two had chromosomal rearrangements resulting in diminished BCL11B expression (data not shown) (Figure 2A).

## Discussion

In this study, we report a novel *de novo* frameshift pathogenic variant (Gly783Ala fs*24) in the *BCL11B* gene associated with craniosynostosis and developmental delay. Using the newly reported AlphaFold algorithm, a computational method that predicts protein structures with atomic accuracy (15), we predicted that this truncated protein would lack the last three C2HH zinc-finger domains (ZnF4-6). As BCL11B is a transcriptional activator, the premature stop-codon sequence of *BCL11B* may affect the protein’s function in binding to its target DNA and its interactions with target proteins (7, 16). We curated the variant as pathogenic based on the ACMG criteria (PVS1_Strong + PS2 + PM6 + PM2). Although we predicted this frameshift variant escape NMD, whether the pathogenic mechanism of the variant is haploinsufficiency or gain of function remains unclear, and further experiment would be needed to address this. In the literature (17), the Cys81Leufs*76 variant was predicted to activate NMD, while the Gly820Alafs*27 variant was predicted to result in a protein with loss of DNA binding to the last C-terminus. Therefore, further studies are needed to elucidate the pathogenic mechanism of *BCL11B* gene.

In addition, this frameshift maybe a pathogenic hotpot of *BCL11B* gene as a second patient who carried the same frameshift mutation was recently submitted in the ClinVar database. Like our patient, this patient presented global developmental delay and the dysmorphic feature like trigonocephaly. However, no more detail clinical information was recorded in this patient. In our patient, we noticed that the number and percentage of eosinophils and the IgE values of our patient significantly increased. Interestingly, five cases with a high level of eosinophils were also reported by Lessel et al and Lu et al. (17, 18). Lu and associates proposed this germline BCL11B-related atopic disease as a novel primary atopic disorder (PAD). The immunophenotyping of the patient revealed intact T-cell numbers as our patient presented (Supplementary Table 1). However, zebrafish experiment demonstrated that the *BCL11B* variant (p.Cys826Tyr) was unable to rescue T-cell development. Therefore, it would be important to explore how immune deficiency arose despite intact thymic development.

In a summary of the neurodevelopment disorder phenotype of all reported patients (8, 9, 17–23), four were reported to present with abnormal MRI, exhibiting moderate ectopia of the amygdala (17), hypoplasia of the globus pallidus (17), callosal agenesis (8), or an abnormal myelination pattern of white matter (23). Our patient had brain morphological abnormalities with brain asymmetry and affected growth of the right brain.

In this study, we reviewed the clinical phenotype of the reported patients with craniosynostosis and *BCL11B* variants (9, 24) and our patient (Table1). One *de novo* missense variant (c.7C > A, p. Arg3Ser) with craniosynostosis in *BCL11B* gene has been reported (9). The patient presented with sever craniosynostosis and received three corrective surgeries before the age of 13 years. Fortunately, the boy was otherwise healthy. Recently, another literature has reported four *BCL11B* missense mutations (p.Gly667Glu, p.Gly582Ser, p.Pro673Arg, p.Pro422Leu) with craniosynostosis or combined congenital diaphragmatic hernia (CDH) and craniosynostosis (24). All the *BCL11B* variants were inherited from their normal parents. The variant of c.1744G > A (p.Gly582Ser) was recorded in three individuals without craniosynostosis in our internal database and in one individual in gnomAD database. In this literature, clinical exome sequencing was performed in one patient and targeted sequencing of single gene (*BCL11B*) in other three patients, these sequence methods may miss real pathogenic gene variants. Therefore, the pathogenicity of these inherited missense *BCL11B* variants in craniosynostosis is questionable as the paper’s title mentioned (24).

## Conclusion

In this study, our data revealed disruptions of *BCL11B* as a monogenic cause of craniosynostosis combined with a neurodevelopmental disorder. Our findings expand the genetic and phenotypic spectrum of the *BCL11B* gene in pediatric patients.

## Data availability statement

The datasets presented in this study can be found in online repositories. The names of the repository and accession number(s) can be found here: the GSA repository, accession number: subHRA004434.

## Ethics statement

The studies involving human participants were reviewed and approved by the ethics committees of the Children’s Hospital, Fudan University (2015-130). Written informed consent to participate in this study and for the publication of any potentially identifiable images or data included in this article was provided by the participants’ legal guardian/next of kin.

## Author contributions

XZ and BW contributed to the conception and design, data acquisition and analysis, and drafting of the manuscript. HC and YW performed the *in silico* analysis. PZ and YQ performed the data analysis of next-generation sequencing. GL performed the genetic experiment and sanger sequence analysis. XP collected clinical information and recorded the data. XD confirmed the clinical diagnosis and silico analysis. HW and CD contributed to the conception, design of this study, and revised the final manuscript. All authors contributed to the article and approved the submitted version.
