# Supplementary material for: Case report: A novel truncating variant of BCL11B associated with rare feature of craniosynostosis and global developmental delay
Source: Front Pediatr. 2022 Oct 6;10:982361. doi: 10.3389/fped.2022.982361 (PMC9582536; doi:10.3389/fped.2022.982361)
Supplement: Supplementary file 1 [file Data_Sheet_1.docx]

**Supplemental Material**

**Supplementary Table 1.**

Immunophenotype of the patient at 25 months

| Index | Value | Normal range |
| --- | --- | --- |
| T cells (%) | 65.51 | 53.88-72.87 |
| Th cells (%) | 36.65 | 24.08-42.52 |
| CD8^+^ T cells (%) | 22.81 | 19.00-32.51 |
| TCRαβ^+^ DNT (%) | 0.53 | 0.37-1.80 |
| γδT cells (%) | 8.12 | 4.94-17.98 |
| CD4^+^ naïve T cells (%) | 72.63 | 46.14-84.40 |
| CD4^+^ central memory T cells (%) | 23.21 | 13.88-48.12 |
| CD4^+^ effector memory T cells (%) | 3.66 | 0.94-6.46 |
| CD4^+^ terminal T cells (%) | 0.50 | 0.00-1.36 |
| CD8^+^ naïve T cells (%) | **87.76↑** | 36.80-83.16 |
| CD8^+^ central memory T cells (%) | 7.59 | 5.18-31.66 |
| CD8^+^ effector memory T cells (%) | 1.40 | 0.70-11.22 |
| CD8^+^ terminal T cells (%) | 3.25 | 0.84-33.02 |
| CD19^+^ B cells (%) | **25.14** | **13.23-26.39** |
| Naïve B cells (%) | **82.51** | **65.54-86.62** |
| Marginal zone B cells (%) | **7.01** | **2.70-19.80** |
| Memory B cells (%) | **6.07** | **2.98-14.18** |
| Transitional B cells (%) | ****3.88↓**** | **5.24-17.22** |
| Plasmablasts (%) | ****0.03↓**** | **0.50-7.06** |
| Eosinophils (%) | **13.7↑** | 0.5-5 |
| Eosinophils (cells/µl) | **910↑** | 60-300 |
| IgE (IU/ml) | **944.91↑** | ＜100 |

Abnormal values are presented in bold and marked with arrows.


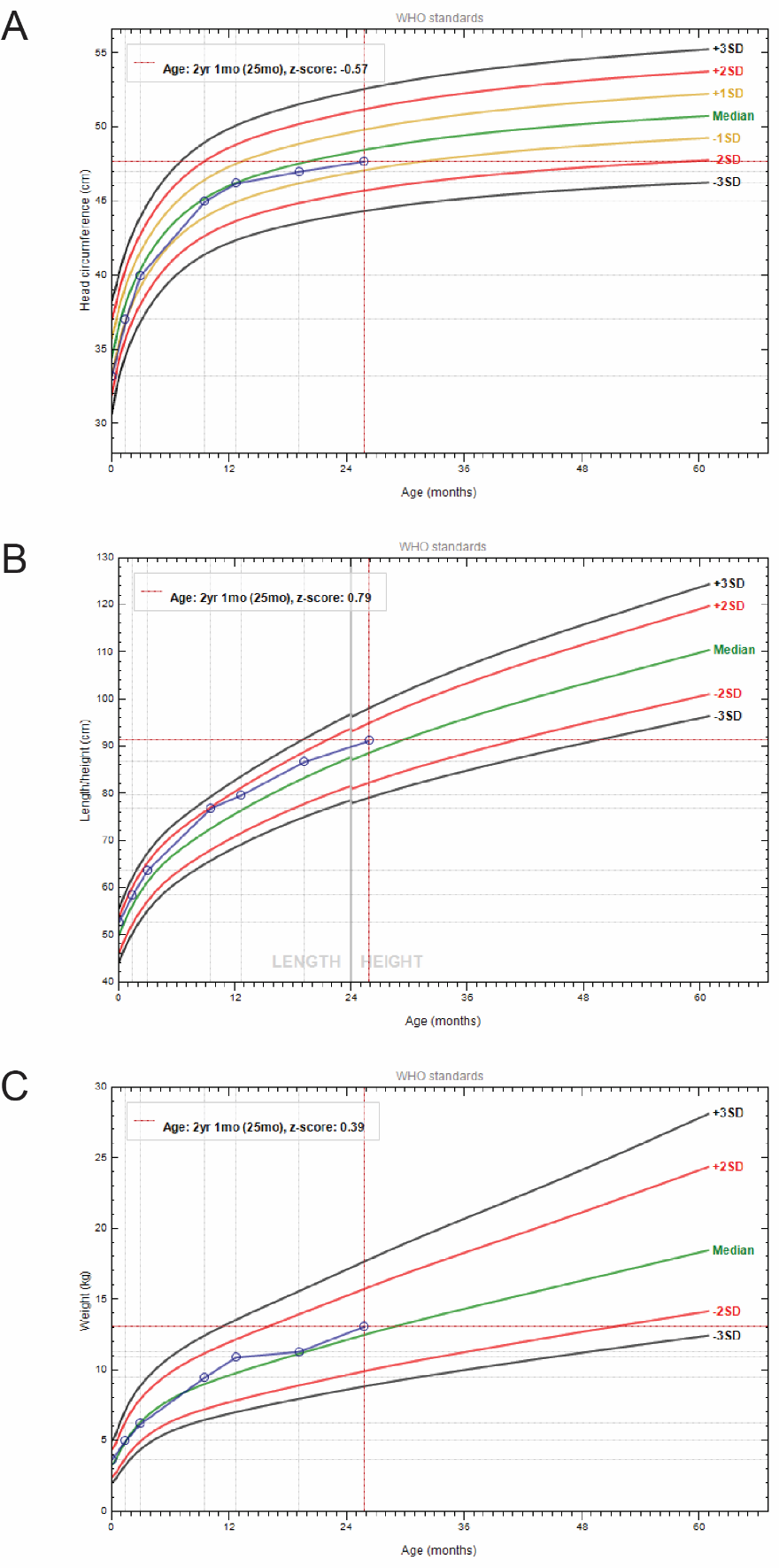
**Supplementary Figure 1.**

**Supplementary Figure 1.** The physical examination records and corresponding SD scores of the patient, according to WHO standards, from birth to 25 months. A. Head circumference of the proband. B. Length/height of the proband. C. Weight of the proband.

**Supplementary Figure 2:**

**
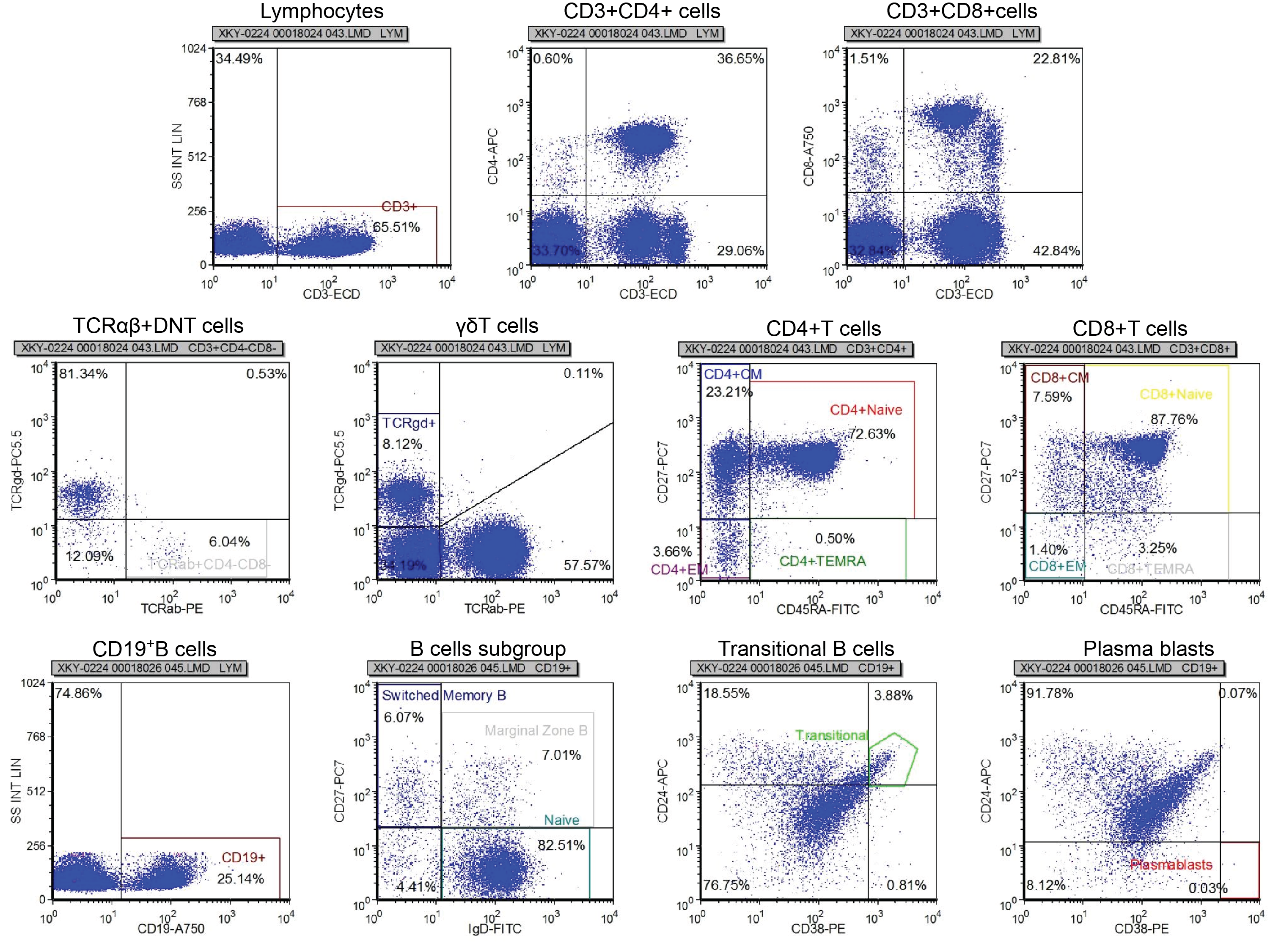
**

**Supplementary Figure 2:** Immune cell analysis of the patient indicated lymphoma. TCRαβ^+^DNT cells were defined as CD3^+^CD4^-^CD8^-^TCRαβ^+^, naïve CD4+/CD8+ T cells were defined as CD3^+^CD4^+^CD27^+^CD45RA^+^/CD3^+^CD8^+^CD27^+^CD45RA^+^, memory T cells were defined as CD3^+^CD45RA^-^, and terminal T cells were defined as CD3^+^CD27^-^CD45RA^+^. Naïve B cells were defined as CD19^+^CD27^-^IgD^+^, marginal zone B cells were defined as CD19^+^CD27^+^IgD^+^, memory B cells were defined as CD19^+^CD27^+^, transitional B cells were defined as CD19^+^CD38^high+^ CD24^high+^, and plasmablasts were defined as CD19^+^CD38^high+^.
